# Recent updates on correlates of vaccine-induced protection

**DOI:** 10.3389/fimmu.2022.1081107

**Published:** 2023-01-27

**Authors:** Stanley A. Plotkin

**Affiliations:** ^1^ University of Pennsylvania, Philadelphia, PA, United States; ^2^ Consultant, Doylestown, PA, United States

**Keywords:** dengue, Ebola, influenza, pneumococcal, respiratory syncytial virus, rotavirus, shigella, tuberculosis

## Abstract

Correlates of protection are key for vaccine development against any pathogen. In this paper we summarize recent information about correlates for vaccines against dengue, Ebola, influenza, pneumococcal, respiratory syncytial virus, rotavirus, shigella, tuberculosis and Zika virus.

## Introduction

A correlate of protection (CoP) is an immune function that correlates with and may be biologically responsible for vaccine-induced efficacy. The literature on this subject has grown considerably since it was identified as an important issue in vaccinology (1–5). The importance of CoP with regard to vaccines against SARS-2, the coronavirus causing COVID-19, needs no emphasis, and numerous papers have been published on that subject (6). However, not so much has been published recently about vaccines against other diseases. This paper is an effort to summarize recent findings in a number of important examples.

It should be acknowledged that the subject of CoP has become more complex due to increasing knowledge concerning Fc Effector antibody mediated functions and T cell mediated functions (7, 8). However, while it is evident that CoPs are often multiple and synergistic, their utility depends on identifying responses that are major and measurable. The fact that immune responses are often synergistic does not negate the value of identifying the main immune function that correlates with the protection generated by vaccination.

The SARS-2 novel coronavirus has been with us for only the last two years, but much work has been expended on defining a CoP, as recently summarized (6). The chief CoP is clearly neutralizing antibodies, with a gradual increase in efficacy as the titer increases. Although T cell responses and Fc effector antibodies are important in modifying the results of infection, antibodies play the major role in preventing infection in the first place (9–11). However, antibodies must be specific for the variant virus, as the neutralizing epitopes differ between strains (12).

## Dengue viruses

The correlates of protection against the four serotypes of dengue virus remain debatable, despite the development of several vaccines that have demonstrated some degree of efficacy (13–17). That efficacy has been influenced by age of the vaccinated population and serotype of the circulating dengue virus. It appears that efficacy is related to the induction of homotypic antibodies, whereas heterotypic antibody may actually enhance disease caused by other serotypes. The Dengvaxia vaccine is licensed only for those aged 9 years or above, and is most effective against dengue serotype 4, against which it induces homotypic specific antibodies. However, in a trial conducted in subjects aged 9 to 16 years with evidence of prior infection with a single serotype, efficacy was 67%, 67%, 80% and 89% respectively against types 1, 2, 3, and 4. Thus, homotypic neutralizing antibodies was the best correlate of protection against infection, but once an individual had infection by one serotype the Dengvaxia vaccine gave efficacy against other serotypes (18, 19). A live attenuated vaccine developed by Takeda was shown to induce that type of response (20). FC effector antibody functions may have a role in protection (21), and antibodies to NS1 reduce severity of disease (14) although no absolute correlate is available. However, at this point the best correlates of protection appears to be type-specific neutralizing antibodies (19).

## Ebola virus

Vectored vaccines against the Ebola filovirus have been highly successful in controlling outbreaks of the disease in Africa. All of the vaccines are able to induce antibodies to the glycoprotein that is present in quantity on the elongated virus particle. However, the CoP is more complex than antibodies alone, and there is strong evidence that CD8+ T cells reacting against Ebola virus are necessary for high efficacy. Thus, Ebola is an example of where both arms of the immune system must respond in order for a vaccine to optimally prevent disease (22–28).

The functions of immune responses induced by successful Ebola vaccines are multiple, involving antibodies to the glycoprotein but also T cell responses (29). The role of the latter in protection appears to depend on the host species, being more important in infections occurring in primates (30). Study of vaccinated subjects also suggests important synergies between antibody and cellular immune functions. Moreover, the selection of adjuvant influences the mechanism of protection (31). For example, a CpG adjuvant stimulating the TLR-9 receptor gave superior survival. In summary, IgG antibody to the viral glycoprotein is the major CoP for Ebola, but is influenced by the type of adjuvant used. In addition, the sheer quantity of glycoprotein on the elongated virus particle may influence the quantity of responses correlated with protection (22, 32–38).

## Influenza

The CoP for influenza that is commonly accepted for influenza is a 1/40 hemagglutinin-inhibition titer, which is credited with signifying a 50% protective ability. This is an oversimplification and ignores many other immunological functions that contribute to the efficacy of influenza vaccines (24, 39). Age of the vaccinee and the type of immunogen also influence the CoP. The widely used HAI titer of 1/40 corresponds to about 50% efficacy in young adults who have had immunologic priming by prior influenza infections. However, that titer conveys lower efficacy in older adults. The single radial hemolysis assay of >25 mm corresponds to about 70% efficacy in adults. In children who have not had prior infection or vaccination an HI titer of 110 gives 50% protection. In any case, in adults protection rises with HI titers, but protection is not guaranteed at higher titers nor absent at lower titers (24, 40).

Although neutralization is clearly an important function of antibodies, Fc effector functions such as antibody-dependent cellular cytotoxicity (ADCC) and antibody-dependent phagocytosis (ADP) play a role in protection. In addition, influenza vaccines contain neuraminidase, though often unmeasured, which contributes to protection (25, 26). Neuraminidase concentration may vary between strains (41). Finally, cell-mediated immunity has not received enough attention and probably contributes to controlling virus replication (42). In summary, multiple antibody functions contribute to influenza vaccine efficacy (43–46).

## Pneumococci

The vaccines against pneumococcal disease are composed of pneumococcal capsular polysaccharides conjugated chemically with proteins to increase immunogenicity, especially in children. For many years an antibody response measured by ELISA with a level of 0.35 micrograms/ml was accepted as a CoP. However, a seminal paper has demonstrated that the protective level is very much dependent on serotype, with type 3 being the most resistant; types 1, 7F, 19A, and 19 F requiring high antibody levels; and types 6A, 6B, 18C and 23F being less resistant (47). Thus, 0.35 mcg gives only a general estimate of a CoP with little precision. For type 3 relatively high levels of antibody are needed, estimated to be 2.83 mcg/ml. This means that the efficacy of conjugated pneumococcal polysaccharide vaccines will vary with the epidemiology of serotypes, and that vaccines will vary in efficacy depending on the composition of serotypes and the sites of infections. The variability of CoPs for different serotypes was recently confirmed by the results of a study in African toddlers that gave 0.26 mcg/ml as CoP for type 14 but 1.93 mcg/ml as CoP for type 23F (28). The conclusion must be that the CoPs for pneumococcal serotypes are variable and must be determined individually.

## Respiratory syncytial virus

Protection against RSV lower respiratory illness is complex: There are two distinct syndromes, one occurring in young infants who have only transplacental neutralizing antibodies to RSV, and a second occurring in the elderly, in whom the pathogenesis of disease is more complex. Antibodies having high neutralizing function are clearly protective in the very young, as shown by the correlation between antibody titer and protection, as well as the prophylactic value of administered monoclonal antibodies (23, 43, 44). A group A RSV inhibitory titer of 1/239 and a group B RSV inhibitory titer of 1/60 were associated with protection against disease (45). Antibodies against the prefusion form of the F protein are those that correlate best with protection. However, Fc effector as well as neutralizing functions of antibody are important, particularly in the lower respiratory tract (23, 34, 46, 47).

On the other hand, pathogenesis of RSV disease is less clear in seropositive elderly adults, in whom administration of antibodies is less effective. In part this may be due to the need to direct antibodies against other antigens of the virus and to elicit functions other than neutralization, particularly T cell functions (35). Antibodies and T cell responses against the small hydrophobic (SH) protein appear to be more important in adult infections. Thus, the problem of RSV vaccine development is less for infants, in whom monoclonal antibodies are protective. The level of neutralizing antibodies in infants predict protection from RSV (45) and thus the problem of vaccine development in infancy could be solved by developing a vaccine based on the prefusion form of the F protein (36, 37). Previously, numerous attempts to develop an RSV vaccine for the elderly have failed to give high levels of protection, despite the use of many strategies including nanoparticle, subunit, live-attenuated and vector-based (36–38, 48). However, the use of prefusion forms of the fusion protein rather than the post-fusion form has recently given encouraging immune responses in adults (49). Although it is uncertain as to whether the efficacy relates to serum or mucosal antibody responses. In addition, cell-mediated immunity may be important for protection of adults (50). However, a monoclonal antibody against prefusion F was successful in preventing RSV disease in children (51).

## Rotavirus

Rotavirus vaccination has been spectacularly successful in high-income countries, though less so in poor countries where children are exposed to many pathogens soon after birth. Over the years since introduction of rotavirus vaccines, an intestinal IgA response and its surrogate, serum IgA, has been considered to be the principal CoP (52–54). A level of more than 20U/ml has been proposed as the protective level (55). However, other studies have not found serum IgA to be a convincing CoP, particularly in low-income countries (56, 57). A thorough review by Clarke and Desselburger (57) concluded that VP6 antibodies may be a better correlate. VP6 is part of the capsid of rotaviruses, and although it does not induce neutralizing antibodies, non-neutralizing antibodies to VP6 develop after infection or vaccination and thus may be a good correlate for protection. However, attempts to develop parenteral vaccines against rotavirus have so far failed, and it appears that secretory responses at the level of the intestine are the best correlates. However, serum IgA serves as an indicator of IgA responses in the intestine (52, 58).

## Shigella

In a review published in 2007 Levine et al. (59) wrote that “Identification of protection is arguably the most crucial catalyst needed to accelerate the development of effective Shigella vaccines,” but added that no clear correlate had been identified. Multiple candidate vaccines against shigella continue to be studied, including those containing the surface O antigen, antibodies to which are one proposed correlate (60, 61). In a detailed analysis Clarkson et al. (62) conclude that there are multiple CoPs, which may differ from one species to another. It appears that both serum and mucosal responses may serve as CoP depending on the challenge situation. This may simply reflect a situation in which the shigella organism must first replicate in the intestine by overcoming mucosal antibodies, but then invade the intestine, where systemic antibodies may be more important. Nevertheless, serum antibodies measured in various ways correlate with efficacy of shigella vaccines (63).

## Tuberculosis

Bacille Calmette-Guérin, an attenuated *Mycobacterium bovis*, has been used for many years as a vaccine against TB, but with efficacy largely confined to vaccination at birth. Many attempts have been made to improve on BCG, for which identification of a CoP would be key. Studies in cows confirm that protective immunity correlates with a Th1 bias and induction of interferon gamma producing T lymphocytes. The presence of central memory T cells also correlates with protection (64). Intravenous BCG given to macaque monkeys also protected against active tuberculosis, which correlated with induction of T cells reacting to tuberculosis antigens (65).

The search for an easily administered and more effective vaccine against human tuberculosis continues. There is agreement that T cells, both CD4+ and CD8+, are key to protection particularly with regard to interferon secretion, but Th17 cells may also play a role. Vaccine delivery by an aerosol route might be preferable (66). A recent review concluded that BCG is only effective in children (67). In any case, it is likely that a T cell function that has not yet been identified will provide the best correlate of protection against tuberculosis (68).

## Zika

As Zika virus is transmitted by mosquito bite, it is not surprising that antibodies in the blood stream are protective. In macaques neutralizing antibody titers of about 1/100 induced by inactivated virus vaccines were shown to be highly protective (69, 70). However, cross-reactive antibodies with other flaviviruses raise questions about whether inducing Zika antibodies might enhance their replication (71).

## Summary

Knowledge concerning correlates of protection by vaccines is critical to their application and continues to grow (5). In this article we report some recent findings for selected vaccines. Although from a biological point of view vaccines produce a variety of protective functions, some are more important than others, and are useful to predict efficacy. **Table 1** lists correlates of protection for some major vaccines.

**Table 1:** Selected correlates of protection after vaccination.

Vaccine	Immune Function	Protection Level
**Anthrax**	Toxin Nt Ab, Anti-PA IgG	1/3000, 10 µg/mL
**Diphtheria**	Toxin Nt Ab	0.01-0.1 IU/mL
***H. influenzae* conjugate**	ELISA Ab	0.15 ng/mL
**Hepatitis A**	ELISA Ab	20 m1U/mLl
**Hepatitis B**	ELISA Ab	10 mlU/mL
**Influenza, inactivated**	HI Ab	1/40 = 50% protection 1/320 in children
	NtAb	1/40 = 50% protection
**Lyme**	ELISA Ab	1400 U/mL
**Measles**	ELISA Ab	≥120 miU/mL
**Meningococcal**	Bactericidal Ab	≥1/4
**Pneumococcal, conjugated**	ELISA Ab	0.20-0.35 µg/mL
**Polio, inactivated**	Nt Ab	≥1/8
**Rabies**	Nt Ab	≥0.5 IU
**Tetanus**	Toxin Nt Ab	0.01-0.1 IU/mL
**Tick-borne encephalitis**	Nt Ab	≥1/10
**Yellow Fever**	Nt Ab	≥0.7 LNI

Current interest in correlates has been raised by the SARS-2 new coronavirus vaccines. As discussed elsewhere (6), the principal correlate of protection is antibodies measured by neutralization or ELISA. However, although there is no threshold value for protection, titers of approximately 1/100 give efficacy against disease better than 50%, whereas titers of 1/1000 or more give efficacy over 90%.

## Data availability statement

The original contributions presented in the study are included in the article/supplementary materials. Further inquiries can be directed to the corresponding author.

## Author contributions

The author confirms being the sole contributor of this work and has approved it for publication.
